# Targeting TLR-4 with a novel pharmaceutical grade plant derived agonist, *Immunomax®*, as a therapeutic strategy for metastatic breast cancer

**DOI:** 10.1186/s12967-014-0322-y

**Published:** 2014-11-29

**Authors:** Anahit Ghochikyan, Alexey Pichugin, Alexander Bagaev, Arpine Davtyan, Armine Hovakimyan, Amir Tukhvatulin, Hayk Davtyan, Dmitry Shcheblyakov, Denis Logunov, Marina Chulkina, Anastasia Savilova, Dmitry Trofimov, Edward L Nelson, Michael G Agadjanyan, Ravshan I Ataullakhanov

**Affiliations:** Department of Molecular Immunology, Institute for Molecular Medicine, 16371 Gothard Street, Suite H, Huntington Beach, CA 92647 USA; The Institute of Immunology, Federal Medical-Biological Agency, Moscow, 115478 Russia; Gamaleya Research Institute for Epidemiology and Microbiology, Russian Ministry of Health Moscow, Moscow, 123098 Russia; Department of Molecular Biology & Biochemistry, University of California, Irvine, CA 92697 USA; Department of Medicine, Division of Hematology and Oncology University of California, Irvine, CA 92697 USA; The Institute for Memory Impairments and Neurological Disorders, University of California, Irvine, CA 92697 USA

**Keywords:** *Immunomax*®, TLR-4 agonist, 4T1 post-resection metastatic breast cancer model, Immunosuppression, Window of opportunity for immunotherapy

## Abstract

**Background:**

Previously we demonstrated that the resection of primary 4T1 tumors only slightly prolongs mouse survival, but importantly, creates a “window of opportunity” with attenuated suppressor cell and increased activated T cell populations. This suggests that additional activation of the immune system by immunostimulatory agents during this period may enhance anti-tumor immunity and potentially eradicate micro-metastatic disease in this stringent model.

We hypothesized that the immunostimulator *Immunomax*®, which is comprised of a plant-derived polysaccharide, is non-toxic in humans and stimulates immune defense during the infectious diseases treatment, may have also anti-tumor activity and be beneficial in the adjuvant setting when endogenous anti-tumor responses are present and during the “window of opportunity” in post-resection metastatic breast cancer model. Here we provide the initial report that *Immunomax*® demonstrates the capacity to eliminate micro-metastatic disease in the post-resection, 4T1 mouse model of breast cancer.

**Methods:**

The efficacy of *Immunomax*® was evaluated by analyzing survival rate and the number of spontaneous clonogenic tumor cells in the lung homogenates of mice. The frequencies of activated NK, CD4^+^ and CD8^+^ cells as well as myeloid-derived suppressor cells and Treg cells were evaluated using flow cytometry. Highly purified mouse and human dendritic and NK cells were sorted and the effect of *Immunomax*® on activation status of these cells was assessed by flow cytometry*.* The property of *Immunomax*® as TLR-4 agonist was determined by NF-κB/SEAP reporter gene assay, WB, RT-PCR.

**Results:**

*Immunomax®* injections significantly prolonged overall survival and cured 31% of mice. This immunostimulator activates DCs via the TLR-4, which in turn stimulates tumoricidal NK cells and *in vitro*, completely inhibits growth of 4T1 cells. Incubation of PBMC from healthy donors with *Immunomax®* activates NK cells via activation of plasmacytoid DC leading significantly higher efficacy in killing of human NK-target cells K562 compared with non-treated cells.

**Conclusion:**

This is the first demonstration that *Immunomax*® is a TLR-4 agonist and the first report of a documented role for this pharmaceutical grade immunostimulator in augmenting anti-tumor activity, suggesting that incorporation of *Immunomax*® into developing breast cancer therapeutic strategies may be beneficial and with less potential toxicity than checkpoint inhibitors.

**Electronic supplementary material:**

The online version of this article (doi:10.1186/s12967-014-0322-y) contains supplementary material, which is available to authorized users.

## Introduction

Breast cancer is the most commonly diagnosed cancer worldwide and it is the second leading cause of death in women [1]. Although, primary treatments (surgery, radiation therapy, and chemotherapy) are beneficial and lead to increased disease free and overall survival, there is a continuous relapse rate that leads to a substantial proportion of breast cancer patients developing recurrent and/or metastatic disease. This indicates persistence of occult microscopic disease that ultimately leads to significant morbidity and mortality (~40,000 deaths in 2011 in the US) despite improved primary and adjuvant treatment. The control of this occult microscopic disease is clearly inadequate and alternative approaches to those currently employed need to be developed. Engagement of the immune system and anti-tumor immune responses is an attractive approach for the control of this occult microscopic, low volume tumor burden, which will ultimately give rise to metastatic lesions. Recent approval of immunotherapeutic products and immune checkpoint inhibitors has reinvigorated the enthusiasm for developing anti-tumor immunotherapeutic strategies. However, the toxicity of checkpoint inhibitors has limited the application of these promising agents in the adjuvant setting, when patients are essentially asymptomatic [2–4]. At the same time there has been limited progress in applying immunotherapy other than adoptive antibody-based therapies (trastuzumab and pertuzumab) to breast cancer, particularly for the control of occult microscopic disease [5–7]. Thus, novel approaches with less toxicity, which have the ability to control occult micro-metastatic disease, are desperately needed to improve survival rates of cancer patients, including breast cancer patients.

Here we investigate the capacity of a potent immunostimulatory compound *Immunomax*® to enhance anti-tumor immunity and control of micro-metastatic disease in the 4T1 post resection metastatic breast cancer model. *Immunomax®* is comprised of a polysaccharide purified from potato sprouts and suspended in a saline [8]*,* it is an injectable pharmaceutical in the Russian Federation (registration P No.001919/02-2002) and 5 other countries of Commonwealth of Independent States (formerly the USSR) and has been evaluated in a wide range of medical situations. In accordance with the formal “Instruction of Medical Use”, one medical indication for *Immunomax*® is the stimulation of immune defense during the treatment of different infectious diseases (http://www.gepon.ru/immax_intro.htm). Thus, we hypothesized that the immunostimulatory activity of *Immunomax*® may be beneficial in the adjuvant setting when endogenous anti-tumor responses are present and during the “window of opportunity”, which we recently identified in the 4T1 post-resection metastatic breast cancer model [9]. In this study, we provide for the first time the initial *in vivo* characterization of the activity and *in vitro* characterization of possible underlying mechanism(s) contributing to the inhibition of metastatic disease and show that pharmacological grade *Immunomax*® is acting as a TLR-4 agonist directly activating DCs and significantly enhancing anti-tumor killing capacity of NK cells.

## Materials and methods

### Mice

Female 8- to 10-wk-old BALB/c mice were purchased from The Jackson Laboratory. All animals were housed in a temperature- and light cycle-controlled facility, and their care was under the guidelines of the National Institutes of Health and the approved Institutional Animal Care and Use Committee protocol at the University of California, Irvine.

### Reagents and antibodies

CD3-PerCP-eFluor710, CD4-eFluor450, CD8-APC, NKp46-PE, FoxP3-FITC, CD69-FITC, rat IgG2a(*κ*)-FITC isotype control, armenian hamster IgG-FITC isotype control, rat IgG2a(*κ*)-PE isotype control, FoxP3 staining buffer set (eBioscience, USA). Gr1-FITC, CD11b-APC (Miltenyi Biotec, USA). Lin1-FITC (CD3, CD14, CD16, CD19, CD20, CD56), CD123-PE, HLA-DR-APC-H7, CD11c V450, CD69-PE, CD56-PE CD45-PerCP, human NK Cell Enrichment Set–DM, CD16-FITC, CD69-PE, CD4-PerCP (BD Biosciences, USA). CD19-FITC, CD11c-PerCP, MHC class II (I-A^d^/I-E^d^)-PE, CD80-FITC, CD86-APC, anti IL-12-PE, Rat IgG2a(*κ)*-APC Isotype Control (BD Pharmingen, USA), CD8a Pacific Blue, CD49b-APC (BioLegend, USA), CellTrace Violet™ Cell Proliferation kit (Invitrogen), CLI-095 (InvivoGen, USA). Pharmaceutical grade *Immunomax®* has been purchased from Immapharma Ltd (Russia).

### Cell lines

Unmodified 4T1 mammary carcinoma cells were obtained from Dr. F.R. Miller (WSU, School of Medicine, Detroit, MI) and were cultured as described in [10]. 4T1-EGFP stable cell line was obtained by a transfection of 4T1 cells with the lentiviral vector pLV-neo-EGFP followed by FACS-sorting.

K562 human erythromyeloid and YAC-1 mouse lymphoma cell lines are from ATCC. HEK-Blue® cells stably transfected with inducible SEAP reporter gene under NF-*κ*B-dependent promoter and human Toll-like receptors 2, 3, 4, 5, 7, 8 or 9, and their co-receptor molecules CD14 and MD2 (HEK-Blue-hTLR2/CD14, HEK-Blue-TLR3, HEK-Blue-TLR4/CD14-MD2, HEK-Blue-TLR5, HEK-Blue-TLR7, HEK-Blue-TLR8, HEK-Blue-TLR9 cells), were obtained from InvivoGen.

### Tumor inoculation

15 × 10^3^ 4T1 tumor cells were injected into the mammary fat pads and tumor growth was monitored daily as described [9,11,12].

### Treatment schedule

**Figure 1:**
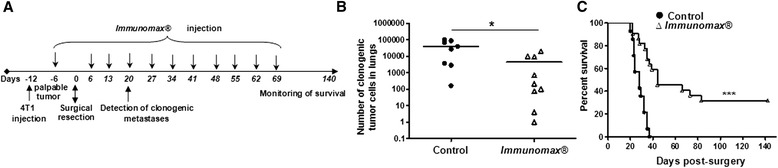
***Immunomax*** **® inhibits metastatic disease and prolongs the survival of 4T1 tumor-resected mice. (A)** Experimental design. **(B)** Three injections of *Immunomax*® significantly decreased the number of clonogenic tumor cells in the lungs of mice (n = 8, control group; n = 9, *Immunomax*® injected group) determined on day 20 post-resection (*P < 0.05). **(C)** Continued weekly injections with *Immunomax*® until the 69^th^ day significantly prolonged the survival of mice (***P < 0.0001, n = 14, control group; n = 22, *Immunomax*® injected group). Thirty one percent of mice from this group were completely cured from the disease. Survival curves were compared using the log rank test with GraphPad Prism.

Mice were injected with 10 μg/100 μl *Immunomax®* (experimental group) or 100 μl PBS (control group) intravenously via the retro-orbital sinuses when tumors are palpable [(approximately 1 mm in tumor diameter (TD)] and once a week after tumor resection. Group of mice were terminated on day 20–22 post-resection, spleens and lungs were harvested for further analysis. Another group of mice received continued injections with *Immunomax®* every week until day 69 and were monitored for survival until day 140 (Figure 1A).

### Surgical removal of tumor and detection of metastases

Tumors were surgically removed when they reached a diameter of 3.5-5 mm. Tumor resection was performed and the number of clonogenic 4T1 cells in the lungs was analyzed as described [9,13,14].

### Isolation of lung mononuclear cells

Mice underwent transcardial perfusion of the lungs with ice-cold PBS before harvesting. Lungs were treated as described in [9].

### Detection of suppressor and effector cell subsets in spleens and lungs

Splenocytes or mononuclear cells from lungs were analyzed with MacsQuant cytometer (Miltenyi Biotec) as we described recently [9].

### GM-CSF induced mouse bone marrow-derived dendritic cells

BALB/c mice bone marrow derived dendritic cells (BM-DC) were differentiated by the induction with GM-CSF during 6–9 days as described [15]. In 6–9 days, the percent of CD11c^+^, I-A/E^+^ cells was around 70-80%.

### Purification of dendritic and NK cells by sorting

Splenic dendritic cells (S-DC) were sorted from the suspension of naïve BALB/c splenocytes as the CD19^−^, CD11c^+^, I-A/E^+^ cells (Additional file 1: Figure S4). BALB/c splenic NK cells were first enriched by EasySep™ Mouse NK Cell Enrichment Kit (STEMCELL Technologies Inc. Canada) then sorted as DX5^+^ I-A/E^−^ cells (Additional file 1: Figure S5A).

Pure BM-DC were sorted from the suspension of 6–9 days culture of GM-CSF derived BM-DC as the CD11c^+^, I-A/E^+^ cells (Additional file 1: Figure S5B).

Human DC were sorted from freshly isolated human PBMC. Plasmacytoid DC (P-DC) were sorted as the Lin1^neg^CD11c^neg^ CD123^high^ HLA-DR^high^ while myeloid DC (M-DC) as the Lin1^neg^CD11c^high^CD123^neg^HLA-DR^high^ ( Additional file 1: Figure S7).

All sorting procedures were performed using BD FACSAria™ II Cell Sorter (BD Biosciences). Purity of sorted cells were 98-99%.

Human NK cells were isolated from PBMC of healthy donor using Human NK Enrichment Set –DM (BD Biosciences) according to manufacturer’s instructions. Purity of the NK enriched cells suspension was 90%.

### 4T1 cells killing assay *in vitro*

4T1 cells were seeded in the wells of 96-well plate at a density of 50 cells/well in 200 μl of complete DMEM and cultured in quadruplicates either alone or in the presence of 5 × 10^4^ splenocytes from naive or tumor-bearing BALB/c mice, and in the presence or absence of *Immunomax®* (10 μg/ml). Cultures were incubated for 7 days at 37°C and 5% CO_2_. 4T1-colonies were fixed using 1% paraformaldehide, stained using 0.5% methylene blue in 50% ethanol. Digital images of each well were taken, then the integrated color density (blue channel) was calculated using ImageJ software (NIH, USA).

In another experimental protocol 200 4T1-GFP cells per a well of 96-well plate were co-cultured with 50000 sorted NK or 25000 sorted BM-DC cells, or both in the presence or absence of *Immunomax®* (10 μg/ml) for 4 days. Number of viable 4T1-GFP cells per well were measured using flow cytometry after PI staining. CLI-095, the inhibitor of TLR4-signaling pathway was used at the concentration 1 μg/ml.

### Immunomax activation of dendritic cells *in vitro*

Sorted S-DC or BM-DC (2 × 10^4^ cells in 200 μl of the complete culture medium) were incubated for 20 hrs at 37°C and 5% CO_2_ in 96-well culture plate in the absence or presence of *Immunomax®* (10 μg/ml). After incubation, cells were stained with CD86-APC or appropriate isotype control and analyzed by FACS.

For intracellular IL-12 staining cells were fixed and permeabilized with BD Cytofix/Cytoperm™ Fixation/Permeabilization Solution Kit then stained with anti-IL12-PE or appropriate isotype control and analyzed by FACS.

Sorted human M-DC and P-DC (0.5x10^3^ cells in 200 μl RPMI-1640 medium supplemented with 10% FCS, 10 mM HEPES, pH 7,4) were stimulated with *Immunomax®* (10 μg/ml) or CpG-2006 (5′-TCGTCGTTTTGTCGTTTTGTCGTT, 5 μg/ml) for 3 hours at 37°C and 5% CO_2_. Cultures were made in triplicates. mRNA was isolated from each culture and expression level for TNF-α, IL-1β and IL-8 was determined by real-time PCR and normalized to the one in the non-stimulated control cultures.

### *Immunomax®* activation of mouse and human NK cells *in vitro*

BM-DCs were obtained as described above. On day 6, *Immunomax®* (10 μg/ml) was added for 18 hrs to obtain the pre-activated BM-DCs. Mouse spleen mononuclear cells were labeled by CellTrace™ Violet and co-cultured with indicated amounts of pre-activated BM-DCs for 4 days. Percent of activated (CD69^+^) and proliferating (CellTrace™ Violet low) CD4^−^,CD8^−^, CD19^−^, DX5^+^ NK cells was measured by flow cytometry.

Enriched human NK cells or fresh blood from the same donor diluted (1:1, v/v) with RPMI-1640 were incubated in the absence or presence of *Immunomax®* (10 μg/ml) for 18 hours at 37°C and 5% CO_2_. Cells were harvested and labeled with the mixture of antibodies CD45-PerCP, CD16-FITC, CD56-PE-Cy™5 and CD69-PE and analyzed on FACS.

### Activation of mouse and human NK cells cytotoxic properties with *Immunomax®*

BALB/c mice were intravenously injected with *Immunomax®* (10 μg) or 0.9% NaCl saline. After 18 hours blood mononuclear cells were isolated using Ficoll-paque (1.09 *g*/cm^3^*)* gradient centrifugation. Co-cultures of PBMC and YAC-1 cells (labeled by CellTrace™ Violet) at different effector/target ratios were incubated for 4 hrs at 37°C and 5% CO_2._

Human PBMC were incubated (18 hours, 37°C, 5% CO_2_) in the absence or presence of *Immunomax®* (10 μg/ml). Co-cultures of PBMC and K562 (labelled by the CellTrace™ Violet) at different effector/target ratios were incubated for 4 hrs at 37°C and 5% CO_2_.

Percent of dead target YAC-1 and K562-cells was measured by flow cytometry after staining with PI (2 μg/ml).

### Determination of TLR-mediated NF-*k*B activation

TLR-dependent activation of NF-*k*B in HEK293-Blue-TLR/CD14-MD2 cells was estimated by determination of SEAP levels in the culture supernatant as previously described [16].

#### Western blot analysis

HEK293-Blue TLR4/CD14-MD2 cells were treated (30 min) with *Immunomax®* (1 μg/ml) or LPS (1 μg/ml), or left untreated. Nuclear proteins were analyzed by Western blotting using anti-p65 and anti-lamin (loading control) antibodies as described [16].

### Statistical analyses

All statistical parameters were calculated using GraphPad Prism 5.0 Software. Statistical significance between groups was determined by two-tailed unpaired ***t*** test (***P*** values less than 0.05 were considered significant) and one-way Anova post Tukey comparison test. Survival curves were generated in GraphPad Prism 5.0 Software and compared using Log-rank (Mantel-Cox) test.

## Results

### *Immunomax*® prolongs the survival of mice following primary 4T1 tumor resection

We recently reported that tumor resection creates a “window of opportunity” (days 3 to 13 post-resection) with increased frequency of CD4 and CD8 positive activated T cells and decreased tumor-associated immune suppression [9]. In the current study, we investigated the efficacy of *Immunomax*® in this model of breast cancer. *Immunomax*® administration was initiated prior to tumor resection, when tumors were just palpable and was continued during the “window of opportunity” (on 6th and 13th days after tumor resection). One group of mice (n = 9) were terminated on day 20 post-surgery and clonogenic tumor cells from metastatic disease in the lungs were analyzed, while the another group of mice (n = 22) continued to be injected with *Immunomax*® every week until day 69 with survival of mice monitored to day 140 (Figure 1A).

On day 20 post-surgery, significantly lower numbers of clonogenic tumor cells were detected in lungs of mice injected three times with *Immunomax*® compared with control animals injected with PBS (Figure 1B). Importantly, continuation of weekly injections of *Immunomax*® resulted in a statistically significant prolongation of survival of mice (***P < 0.0001 by log rank test) and complete cure in 31% of the mice (Figure 1C). On day 37 after surgery when all control mice had died from metastatic disease, 63% of mice injected with *Immunomax*® were still alive. Median survival time was 28 days for the control group and 44 days for *Immunomax*® treated group. This survival data is a significant improvement from previously published results of treatment of primary 4T1 tumor-resected mice with other immunotherapeutic agents [17].

### Increased frequency of activated NK, CD4^+^ and CD8^+^ cells along with decreased frequency of myeloid-derived suppressor cells in primary 4T1 tumor-resected, *Immunomax*® treated mice

**Figure 2:**
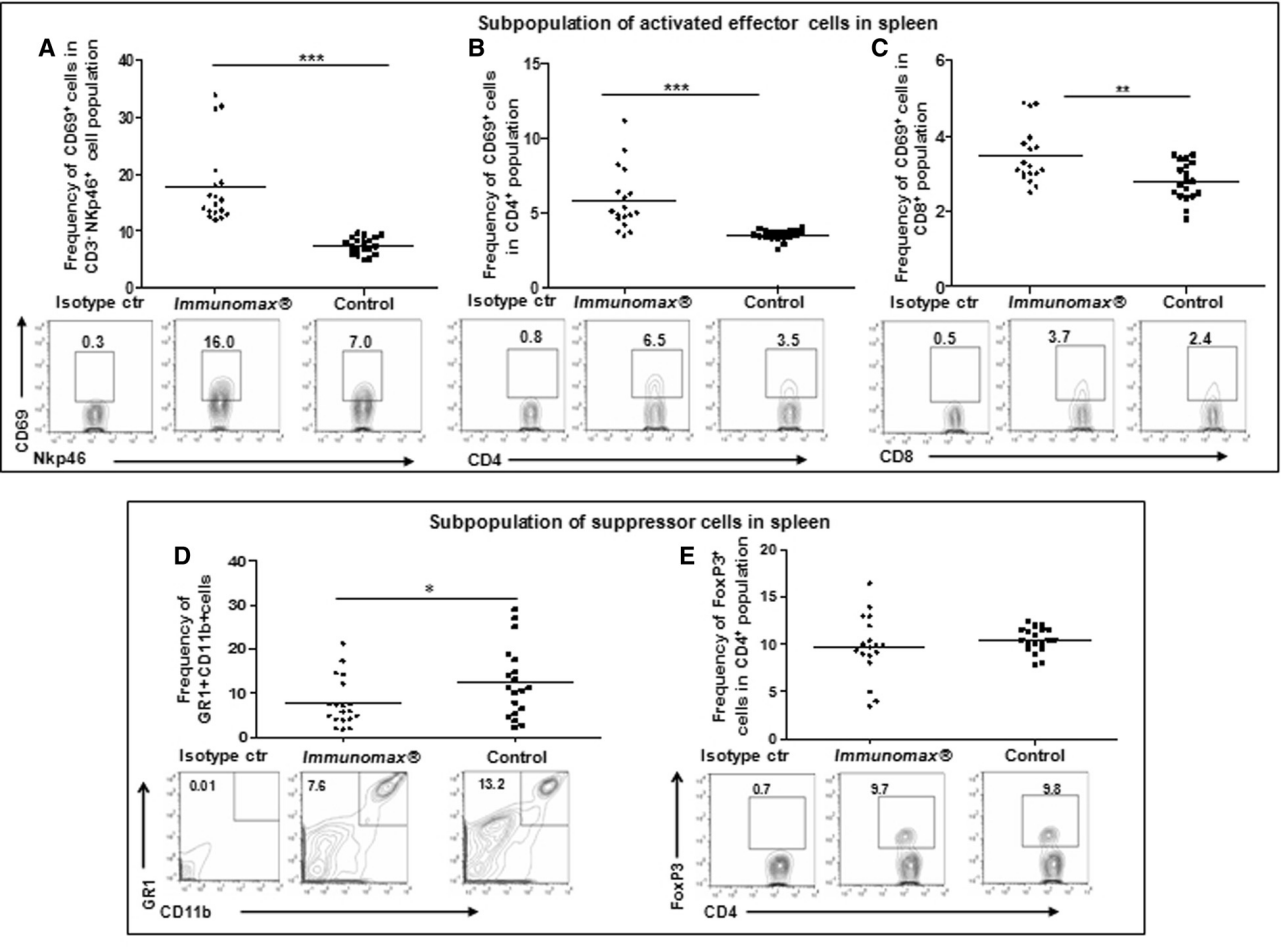
**Effect of** ***Immunomax*** **® on immune effector and suppressor cells.** *Immunomax*® increased the frequency of activated effector cells [NK **(A**, ***P < 0.001), CD4^+^ **(B**, ***P = 0.0001) and CD8^+^ **(C**, **P = 0.0033)] and reduced the frequency of GR1^+^CD11b^+^ MDSC **(D**, *P < 0.05) without changing the levels of Treg cells among CD4^+^ cell population **(E)** in spleens of treated 4T1 tumor-resected mice (n = 18) compared with control mice (n = 19). Representative FACS images are presented. Numbers indicate the percentage of cells in a quadrant.

Given this activity of *Immunomax*®, we decided to evaluate possible modulation of different subsets of immune cells [activated NK, CD4^+^, CD8^+^ cells as well as myeloid-derived suppressor cells (MDSC) and regulatory T cells (Treg)] in both the spleen and lungs of mice treated with *Immunomax*®. Significantly higher percentage of CD69^+^ cells were detected in splenic NKp46^+^ NK (17.78 ± 1.59% vs 7.47 ± 0.33, P < 0.0001, Figure 2A), CD4^+^ (6.19 ± 0.53% vs 3.88 ± 0.14% ***P = 0.0001, Figure 2B) and CD8^+^ (3.45 ± 0.17% vs 2.79 ± 0.11% **P = 0.0033, Figure 2C) cell populations of *Immunomax*® treated mice compared with PBS injected animals. The frequency of MDSC was slightly but significantly decreased in the spleens (7.86 ± 1.26% vs 12.43 ± 1.84%, *P < 0.05, Figure 2D), while no changes were observed in the frequency of Treg cells (Figure 2E). We did not observe any significant changes in the frequency of activated NK, CD4^+^, CD8^+^ as well as MDSC and Treg cells in the lungs of these animals, although there was a trend toward increased activated effector NK and CD4^+^ cells along with decreased Treg cells (Additional file 1: Figure S1). As injections of *Immunomax*® contributed to decrease of metastatic disease, prolonged survival and complete cure of 31% of 4T1 tumor-resected animals we suggest that in treated mice the infiltration of effector cells into the lungs may occur before or after the selected time point of 20 days post-surgery, given the data on splenic populations.

### *In vitro* investigation of the possible mechanisms underlying the anti-metastatic activity of *Immunomax*®

The efficacy of *Immunomax*® against 4T1 micro-metastatic disease could potentially be based on a direct inhibitory effect on the growth of 4T1 breast cancer cells. We tested this possibility and did not detect significant changes in growth of 4T1 cells after *in vitro* incubation with or without *Immunomax*®*.* However, addition of splenocytes isolated from tumor-free mice to 4T1 cultures along with *Immunomax*® dramatically inhibited the growth of 4T1 cells (Additional file 1: Figure S2A). This effect was not dependent upon having effector cells that had not been exposed to the tumor growth, as *in vitro* incubation of splenocytes from tumor-bearing mice with *Immunomax*® dramatically inhibited the 4T1 cells*,* as measured by *in vitro* clonogenic assays (Additional file 1: Figure S2B)*.* These data indicate that even in the presence of substantial amount of suppressor cells in the spleens of 4T1 tumor-bearing mice [9,18,19], splenocytes obtained from these animals are still capable of diminishing tumor growth upon activation with *Immunomax*®.

**Figure 3:**
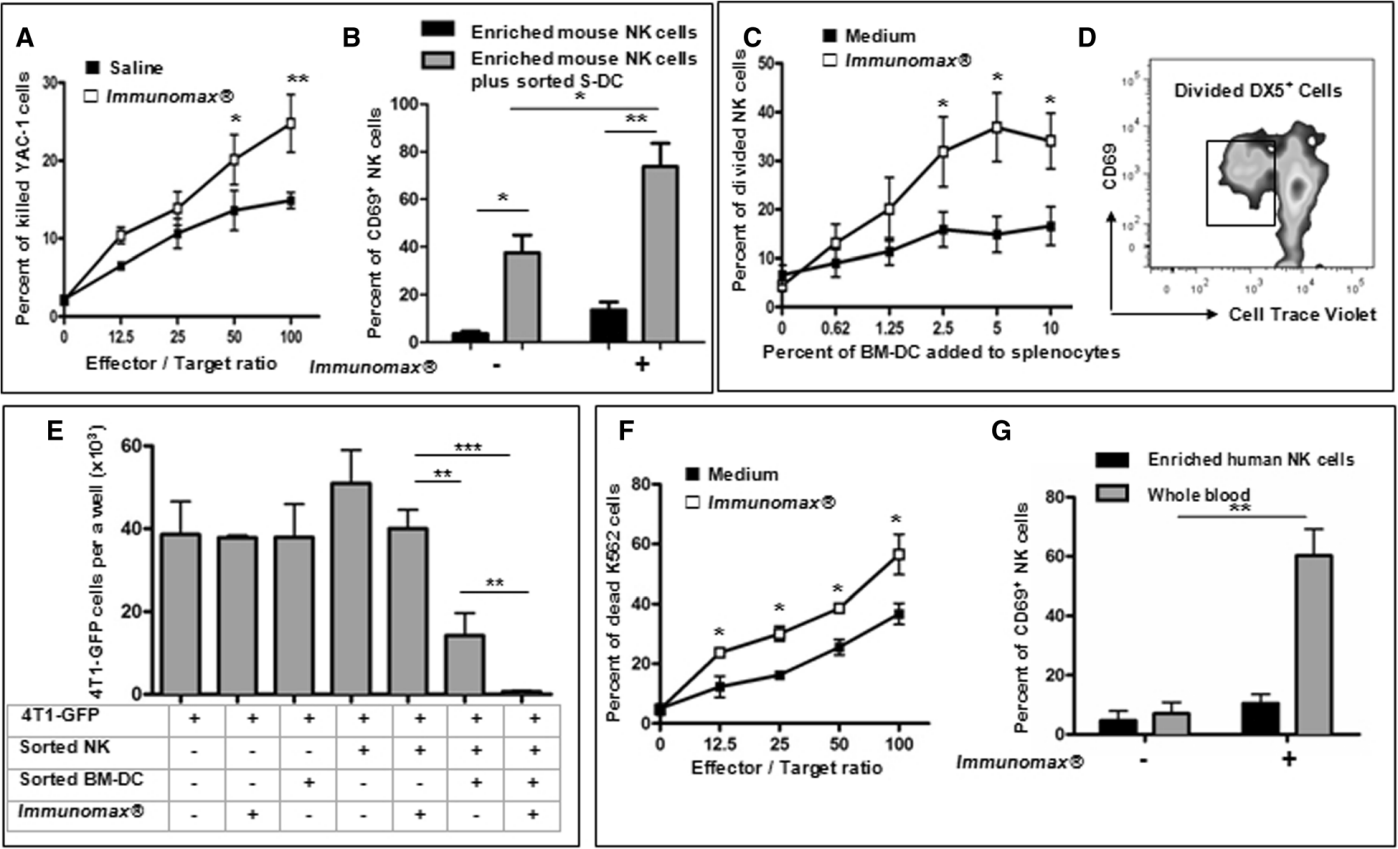
***Immunomax***® **activates mouse and human NK cells and this activation is DC-mediated. (A)** PBMC of mice injected with *Immunomax*® (i.v.,10 μg, □) have significantly higher rate of cytotoxicity against NK-sensitive YAC-1 target cells than PBMC from mice (■) injected with PBS. Consolidated data (mean ± SE) of two independent experiments are presented (*P<0.05, **P<0.01). **(B)** *Immunomax*® significantly increased the frequency of activated NK cells in purified NK cell/S-DC co-culture but not in purified NK cell culture (*P<0.05). **(C)** *Immunomax*® induced proliferation of mouse DX5^+^ NK cells via the activation of BM-DC. BM-DC pre-activated with *Immunomax*® (10 μg/ml, 18 hrs, □) were more effective in activation and proliferation of NK cells than control BM-DC without *Immunomax*® pre-incubation (■). Consolidated data (mean ± SE) of three independent experiments are presented (*p<0.05). **(D)** A percent of activated and proliferating NK cells in CD4^-^, CD8^-^,CD19^-^,DX5^+^ subset of mouse splenocytes. **(E)** Activation with *Immunomax*® significantly increased the ability of mouse purified NK/BM-DC co-culture to inhibit the growth of 4T1-GFP tumor cells (**P<0.01). **(F)** Human PBMC stimulated *in vitro* with *Immunomax*® (10 μg/ml, □) killed K562 cell line more effectively than control PBMC (■) (*p<0.05). **(G)** *Immunomax*® activated NK cells in human whole blood (gray square), but did not activate purified fraction of NK cells from the same blood (■). Consolidated data (mean ± SD) of three independent experiments are represented (**P<0.01).

Previous work has demonstrated that *Immunomax*® stimulates innate immune responses including: production of pro-inflammatory cytokines, production of NO by murine macrophages and human monocytes, and increasing the cytotoxic efficacy of human NK cells [20,21]. These data and our results on modulation of cellular subsets presented in Figure 2 suggest a possible role for *Immunomax*® in activation of mouse NK cells which are generally considered to be a component of the first line of defense against cancer [22]. Indeed results presented in Figure 3A demonstrate that peripheral blood mononuclear cells (PBMC) collected from mice 18–20 hrs after injection of *Immunomax*® have significantly higher cytotoxic activity against YAC-1, a classical mouse NK-target tumor cell line, as compared to PBMC from vehicle only injected control mice. However, *Immunomax*® did not directly activate purified splenic NK cells, but did significantly increase the capacity of S-DC to activate NK cells. The percent of activated NK cells in NK/S-DC co-culture in the presence of *Immunomax*® was significantly higher than without *Immunomax*®*,* (Figure 3B, for gating strategy and purity of cells see Additional file 1: Figure S3 and S4).

Titration of purified mouse BM-DC, pre-activated with *Immunomax*® *in co-culture with* naïve splenocytes, showed that maximum number of CD69 positive activated NK cells was detected when splenocytes were co-cultured with 5% of purified BM-DC (Figure 3C,D**)**. Collectively these data indicate that activation of NK cells by *Immunomax*® is indirect and dependent, at least in part, on the presence of syngeneic myeloid DC. Using *in vitro* 4T1-GFP clonogenic assays we demonstrated that highly purified NK or highly purified BM-DCs (Additional file 1: Figure S5) individually were unable to inhibit 4T1-GFP growth (Figure 3E). However co-cultures of these cells exerted significant anti-4T1-GFP tumor effect (**P < 0.01). Although *Immunomax*® itself had no effect on 4T1-GFP growth, the addition of *Immunomax*® to the NK/BM-DC co-cultures dramatically improved the inhibition of 4T1-GFP growth (***P < 0.001). These data were confirmed with human PBMC stimulated with *Immunomax*®, which killed the human NK target cell line, K562, more effectively than non-stimulated PBMC (Figure 3 F). As in case of mouse NK cells, *Immunomax*® does not directly activate purified human NK cells, but dramatically activates this subpopulation of immune cells in human whole blood (Figure 3G, see gating strategy in Additional file 1: Figure S6). Collectively these data lead us to conclude that *Immunomax*® does not directly activate mouse or human NK cells and activation of this subpopulation of immune cells is mediated by at least a subset of myeloid DCs, which have been described as powerful enhancers of NK cell activity [23–25].

**Figure 4:**
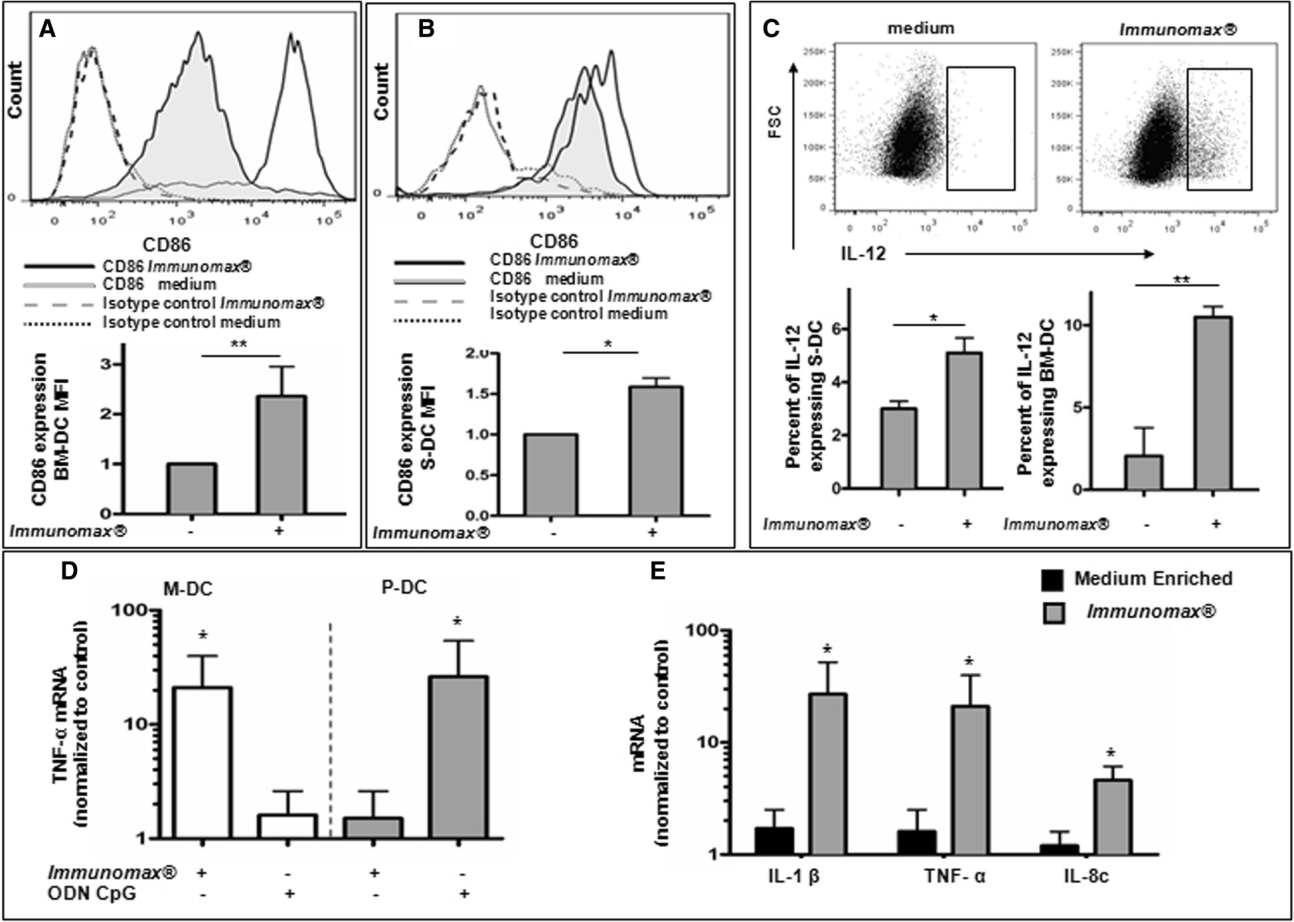
***Immunomax*** **® activates mouse and human dendritic cells. (A,B)** Incubation of Balb/c mouse BM-DC **(A)** and S-DC **(B)** with *Immunomax®* (10 μg/ml) for 18 hrs increases the expression of surface CD86 co-stimulatory molecules. Upper panels depict flow cytometry histograms for CD86 after incubation in the absence (solid line with gray filling) or presence (solid line without filling) of *Immunomax*®, as well as appropriate isotype controls. Lower panels depict normalized mean fluorescence intensity (mean ± SD, **P < 0.01). **(C)** *Immunomax*® increases production of IL-12 cytokine by mouse S-DC and BM-DC. Upper panels depict flow cytometry dot plots for IL-12 expressing DC after incubation in the absence or presence of *Immunomax*®. Lower panels depict percent of IL-12 expressing S-DC and BM-DC (mean ± SD, *P < 0.05, **P < 0.01). Consolidated data of three independent experiments are shown. **(D,E)** *Immunomax*® activates myeloid but not plasmocytoid human dendritic cells. **(D** *TNF-α* mRNA was oppositely expressed in sorted M-DC (lin1^neg^ CD11c^high^ CD123^neg^ HLA-DR^high^) (□) and P-DC (lin1^neg^ CD11c^neg^ CD123^high^ HLA-DR^high^) (gray square) in response to *Immunomax*® or ODN CpG-2006, respectively. **(E)** *Immunomax*® (gray square) increases transcription of genes encoding *IL-1β*, *TNF-α* and *IL-8* in sorted human M-DC relative to untreated control M-DC (■). Consolidated data (mean ± SD) of 7 separate experiments are represented.

In mice and humans there are several types of DC that differ in morphology, localization, lineage, maturation state, function and phenotype. *Immunomax*® induced up-regulation of CD86 co-stimulatory molecule expression (Figure 4A,B) and also increased the percent of purified mouse BM-DC (CD11c^+^/I-A/E^+^) or S-DC (CD19^−^/CD11c^+^/I-A/E^+^) producing the important Th1-type cytokine, IL-12 (Figure 4C). For human DCs purified from PBMC, incubation of *Immunomax*® with M-DC (Lin1^neg^ CD11c^high^ CD123^neg^ HLA-DR^high^), but not P-DC (Lin1^neg^ CD11c^neg^ CD123^high^ HLA-DR^high^), led to up-regulation of *TNFα* mRNA, whereas, the TLR-9 agonist ODN CpG induced up-regulation of *TNFα* mRNA in P-DCs, but not in M-DCs (Figure 4D, see gating strategies and cell purity in Additional file 1: Figure S7). These results were corroborated by up-regulation of transcription of other pro-inflammatory cytokines genes, such as *IL-1β* and *IL-8* by M-DC activated with *Immunomax*® (Figure 4E). Taken together, the combined *in vivo* and *in vitro* data may suggest that *Immunomax*® likely activates DC, which in turn induce the activation of NK cells.

**Figure 5:**
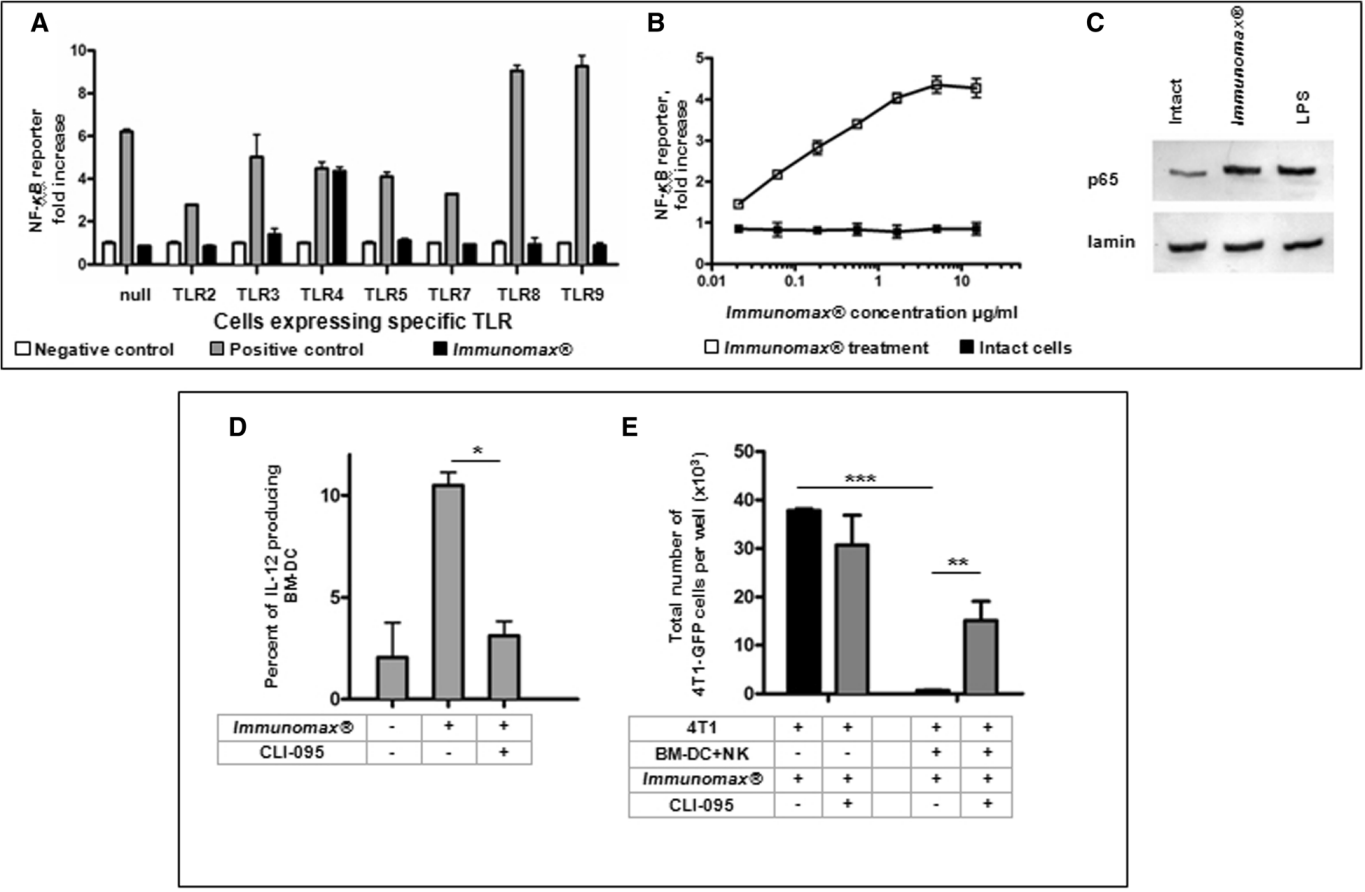
***Immunomax*** **® activates NF-** ***k*** **B via toll-like receptor 4. (A)** NF-*κ*B activity was measured as NF-*κ*B-dependent SEAP reporter gene expression in HEK-Blue TLR null, 2, 3, 4, 5, 7, 8, or 9 cells incubated for 18 hours in the presence (5 μg/ml, ■) or absence (negative control, □) of *Immunomax*®. TNF-α (10 ng/ml), Pam_2_CSK4 (1 μg/ml), poly I:C (10 μg/ml), LPS (1 μg/ml), flagellin (1 μg/ml), Imiquimod (1 μg/ml), CL097 (1 μg/ml) and ODN 2007 (10 μg/ml) were used as positive controls for TLR null, 2, 3, 4, 5, 7, 8 and 9 positive cells, respectively (gray square). Results are expressed as the fold-increase in NF-*κ*B reporter (SEAP) activity relative to intact (untreated) cells. Mean values from 3 independent experiments, each performed in duplicates are presented. **(B)** *Immunomax*® induces NF-*κ*B-dependent reporter (SEAP) gene expression in HEK-Blue TLR-4 cells in a dose-dependent manner. Intact HEK-Blue TLR-4 cells and HEK-Blue TLR null cells were used as negative controls. Results are expressed as the fold-increase in NF-*κ*B-dependent reporter (SEAP) activity in *Immunomax*® treated (□) relative to intact (untreated ■) cells. Mean values from 3 independent experiments, each performed in duplicate are presented. **(C)** NF-*κ*B activation in HEK-Blue TLR4 cells was analyzed according to the nuclear translocation of p65 subunit of NF-*κ*B. **(D,E)** Selective blockade of TLR4-signaling pathway abrogates the induction of DC activation by *Immunomax*®. **(D)** Addition of CLI-095 (10 μg/ml) to the BM-DC/*Immunomax*® culture significantly inhibits the number of BM-DC producing IL-12 cytokine. **(E)** CLI-095 added into the co-culture 4T1/BM-DC/NK cells inhibited the ability of *Immunomax*® to activate BM-DC/NK cell-mediated killing of 4T1 breast cancer cells.

Given the expression of proinflammatory cytokines induced in DC by *Immunomax*®_*,*_ indirect activation of NK cells, and the differential TLR expression patterns in M-DC and P-DC [26], we hypothesized that TLR receptors were involved in the specificity and activity of *Immunomax*®. To evaluate this hypothesis we used a collection of HEK-Blue cell lines with stable expression of individual human Toll-like receptors, i.e. human TLR-2, 3, 4, 5, 7, 8, 9, and the inducible secreted embryonic alkaline phosphatase (SEAP) reporter gene under the control of a NF-*κ*B-dependent promoter (Invivogen). Incubation of these cell lines with *Immunomax*® demonstrated the activation of NF-*κ*B signaling pathway only in hTLR-4 HEK Blue cells (Figure 5A). We detected a dose dependent increase in nuclear NF-κB activation that was saturated at concentrations of *Immunomax*® in the low μg/ml range (Figure 5B, Additional file 1: Figure S8), supporting this as a specific receptor ligand interaction. Additionally, a similar increase in nuclear translocation of the p65 component of the NF-κB complex was observed after stimulation of hTLR-4 HEK Blue cells by either LPS or *Immunomax*® (Figure 5C).

These data suggest that TLR-4 is involved in *Immunomax*® activity, but it remained to be determined if TLR-4 was necessary and sufficient for this activity. We assessed the production of IL-12 by BM-DC incubated in the presence or absence of *Immunomax*® and/or CLI-095, a peptide known to inhibit TLR-4-signaling pathway [27,28] and showed that CLI-095 dramatically inhibited activation of DC (Figure 5D). In addition, we demonstrated that inhibition of TLR-4 signal transduction and its downstream signaling by CLI-095 inhibited killing of 4T1 tumor cells by the mixture of purified BM-DC and NK cells activated by *Immunomax*® *in vitro,* reversing it to the level exhibited in NK/BM-DC co-culture before the addition of *Immunomax*® (Figure 5E). Taken together, these data support that TLR-4 signaling is necessary and sufficient for *Immunomax*® activity.

## Discussion

Recent developments in the field of tumor immunology highlight the necessity of altering the balance between immunosuppressive factors and proinflammatory influences in order to establish and maintain effective anti-tumor immune responses. Our data reported herein identify the immunostimulatory agent, *Immunomax*® as a TLR-4 agonist with anti-tumor activity in the stringent 4T1 murine breast cancer model, which recapitulates human micro-metastatic disease.

In the setting of primary tumor resection, analogous to the human clinical situation, we have demonstrated both improved survival and decreased clonogenic pulmonary metastatic tumor cells with *Immunomax*® administration (Figure 1B,C). *Immunomax*® significantly increased the percent of activated CD69^+^ NK cells, CD69^+^CD4^+^, and CD69^+^CD8^+^ T cells, and decreased the percent of MDSC in spleens of treated mice (Figure 2). This anti-tumor activity of *Immunomax*® appears to be mediated, at least in part, through direct activation of DC and indirect activation of NK cells (Figures 3 and 4). An *in vitro* exploration of the possible mechanism that underlies the *Immunomax*® immunomodulatory properties revealed that this molecule is a TLR-4 agonist (Figure 5), which directly activates DCs (murine S-DC and BM-DCs along with human M-DCs), which in turn are capable of activating NK cells.

TLR-4 signaling in cancer is considered a double-edged sword leading to both cancer inhibition and growth [29] as not only immune cells, but also histologically different tumor cells express different levels of TLR-4. It has been shown that the TLR-4 agonist, LPS, enhances 4T1 tumor growth and metastases, by increasing angiogenesis, vascular permeability and tumor invasion [30], likely through the release of various immunosuppressive cytokines as well as pro-inflammatory cytokines and chemokines in the tumor microenvironment. This effect is more profound in the TLR-4^−/−^ mice suggesting that TLR-4 expressed on host immune cells may play a key role in inhibiting breast cancer progression and metastasis [31]. We also have demonstrated that injection of *Immunomax*® in mice with the large 4T1 tumors fails to inhibit tumor growth and metastatic disease (data not shown)*.* These and other data reviewed in [29,30] suggests that the effect of triggering of TLR-4 agonist is context dependent and may induce pro or anti-tumorigenic effects. In our current study, *Immunomax*® has predominant anti-tumor effects both *in vitro* and *in vivo.* Importantly, this effect in the mouse model of resected 4T1 primary breast tumor, during the “window of opportunity”, when the tumor microenvironment and tumor-associated immunosuppression is diminished, supports the role of this agent in low tumor burden states, i.e. the adjuvant setting.

Indeed, dating back to Coley’s toxins, TLR ligands have periodically been investigated for the treatment of cancer with mixed results, likely due to their intrinsic toxicities and the context in which they have been used. Characterization of the TLR system and identification of specific ligands for the TLRs, has advanced the biological rationale for testing of TLR ligands for treatment of cancer. The safety of LPS, the classic ligand for TLR-4, as a standalone medicine was tested and low doses (4 ng/kg) that are relatively safe were determined in early clinical trials in patients with colorectal cancer, non-small-cell lung carcinoma, renal cell carcinoma, pancreatic cancer, sarcoma, anal, gallbladder and trachea cancer [32]. Although high doses of non-steroid anti-inflammatory drugs such as ibuprofen were administered in parallel to prevent endotoxic shock, transient renal and hepatic toxicities occurred in several patients [32,33]. Currently LPS is under investigation in a clinical trials for anti-cancer therapy [34]. Monophosphoryl lipid A (MPLA) is the less toxic derivative of LPS and included in the ranked NCI’s list of immunotherapeutic agents with the highest potential to treat cancer [35]. Although MPL proved to be safe as an adjuvant, it is unlikely to be approved as a monotherapy for cancer, due to less efficacy while LPS is the best activator of DC but is generally considered to be too toxic for humans. Thus, there remains a void in the TLR-4 agonistic agent repertoire to be filled by less toxic but very active TLR-4 agonists.

*Immunomax*® is a pharmaceutical grade polysaccharide of plant origin that is used for humans as an immune stimulator for correction of a compromised immunity and treatment of different viral and bacterial infections. The safety and non-toxic nature of *Immunomax*® has been demonstrated through its use for the treatment of viral and bacterial diseases in the Russian Federation and other CIS countries over the past decade. During 12-years period of use there was not a single acknowledgement of any side, adverse or toxic effect recorded although the Russian rule requests to immediately inform both the Ministry of Health and the Manufacturer about any of such events. More recently, *Immunomax*® has been successfully tested in Germany in initial studies performed for the treatment of prostate carcinoma and chronic prostatitis IIIa (NIH-CPSI) [21].

It is likely that successful immunotherapeutic strategies will entail strategic utilization of several agents targeting different aspects of anti-tumor immune responses and placing a premium on non-toxic agents. Agents that are immunostimulatory and that modulate the immunosuppressive environment will likely be an important element of successful anti-tumor immunotherapy. The approach known as immune checkpoint blockade (Yervoy, Bristol-Myers Squibb; anti-PD-1 and anti-PD-L1 antibody) was demonstrated to augment anti-tumor immunity and was associated with clinical responses in cancer patients [36–38]. However these and other immunomodulatory drugs cause several immune related adverse events that limit their use. *Immunomax*® activating influence on tumoriсidal properties of DC with their NK cell alliance and its impact on the proportion of various immune cellular subsets in a favor of effector rather than suppressor activity, suggests that administration of this compound for human use may favorably shift the balance of the immune system, potentially without the toxicity of the currently available immune checkpoint inhibitors. The capacity to limit the growth and development of metastatic tumor in a stringent 4T1 breast cancer model makes this novel TLR-4 agonist particularly attractive.

## Conclusion

This is the first demonstration of anti-tumor activity of *Immunomax*® against occult micrometastatic tumor and the first *in vitro* demonstration that this immunostimulator is a TLR-4 agonist directly activating DC and NK cells co-operation. Data presented here suggest that *Immunomax*® could be readily translated into the clinical tumor treatment arena and could result in a new, non-toxic element of immunotherapeutic strategies to improve the control of occult metastatic disease in patients after surgical removal of their primary tumors.

## Additional file

Additional file 1: Figure S1. The level of activated effector [NK cells (A), CD4^+^(B) and CD8^+^T cells (C)] or suppressor [MDSC (D) and Treg (E)] cells in the lungs of Immunomax® injected mice on day 20 post-surgery. Representative FACS images are presented. **Figure S2.** (A) Complete inhibition of 4T1 cell growth by splenocyte/4T1 co-culture in the presence of Immunomax®. (B) Activation of splenocytes isolated from 4T1 tumor bearing mice with Immunomax® inhibited the growth of splenic metastatic tumor cells. **Figure S3.** Flow cytometry gating strategy for CD4^-^, CD8^-^, CD19^-^, DX5^+^ NK cells in splenocytes. **Figure S4.** The gating strategy (A) and purity (B) of sorted population of mouse S-DCs. Purity of the sorted population was 97-98%. **Figure S5.** Purity of sorted splenic NK cell (A) and BM-DC (B) populations. **Figure S6.** A flow cytometry gating strategy to identify NK cells (CD45^+^, CD56^+^, CD16^+^) and representative histograms of CD69- expression by NK cells pre-incubated with or without Immunomax®. **Figure S7.** Human blood DC FACS-sorting (A) and gating (B) strategy. P-DC were identified as the lin1^neg^ CD11c^neg^ CD123^high^ HLA-DR^high^, while M-DC as the lin1^neg^ CD11c^high^ CD123^neg^ HLA-DR^high^. (C) Purity of the sorted M-DC and P-DC. (D) Sensitivity of RT-PCR using the sorted DC. RT-PCR data for mRNA of hypoxanthine-guanine phosphoribosyl-transferase using different numbers of sorted DC per sample are represented. **Figure S8.** NF-κB activity measured as NF-κB-dependent SEAP reporter gene expression in HEK-Blue TLR null, 2, 3, 4, 5, 7, 8, 9 cells treated with Immunomax®. Intact cells were used as the negative control. TNF-α (10 ng/ml), Pam2CSK4 (1 μg/ml), poly I:C (10 μg/ml), LPS (1 μg/ml), flagellin (1 μg/ml), Imiquimod (1 μg/ml), CL097 (1 μg/ml), ODN 2007 (10 μg/ml) were used as positive controls. Results are expressed as the fold-increase in NF-κB-dependent SEAP reporter gene activity relative to intact untreated cells.

## References

[1] American Cancer Society, 2013. **Cancer Facts & Figures.** ACS website: http://www.cancer.org/research/cancerfactsfigures/cancerfactsfigures/cancer-facts-figures-2013.

[2] Gogas H, Polyzos A, Kirkwood J (2013). Immunotherapy for advanced melanoma: fulfilling the promise. Cancer Treat Rev.

[3] Mellman I, Coukos G, Dranoff G (2011). Cancer immunotherapy comes of age. Nature.

[4] Ascierto PA, Simeone E, Sznol M, Fu YX, Melero I (2010). Clinical experiences with anti-CD137 and anti-PD1 therapeutic antibodies. Semin Oncol.

[5] Mittendorf EA, Clifton GT, Holmes JP, Clive KS, Patil R, Benavides LC, Gates JD, Sears AK, Stojadinovic A, Ponniah S, Peoples GE (2012). Clinical trial results of the HER-2/neu (E75) vaccine to prevent breast cancer recurrence in high-risk patients: from US Military Cancer Institute Clinical Trials Group Study I-01 and I-02. Cancer.

[6] Baselga J, Cortes J, Kim SB, Im SA, Hegg R, Im YH, Roman L, Pedrini JL, Pienkowski T, Knott A, Clark E, Benyunes MC, Ross G, Swain SM (2012). Pertuzumab plus trastuzumab plus docetaxel for metastatic breast cancer. N Engl J Med.

[7] Khasraw M, Bell R (2012). Primary systemic therapy in HER2-amplified breast cancer: a clinical review. Expert Rev Anticancer Ther.

[8] Ataullakhonov RI, Melnikova TM, Khaitov RM, Pichugin AV: **Plant extract active as an immunostimulating agent.** United States Patent Application Publication. Pub. No.: US 2005/0042236 A1. Pub. Date: Feb. 24, 2005.

[9] Ghochikyan A, Davtyan A, Hovakimyan A, Davtyan H, Poghosyan A, Bagaev A, Ataullakhanov RI, Nelson EL, Agadjanyan MG (2014). Primary 4T1 tumor resection provides critical “window of opportunity” for immunotherapy. Clin Exp Metastasis.

[10] Vasilevko V, Ghochikyan A, Sadzikava N, Petrushina I, Tran M, Cohen EP, Kesslak PJ, Cribbs DH, Nicolson GL, Agadjanyan MG (2003). Immunization with a vaccine that combines the expression of MUC1 and B7 co-stimulatory molecules prolongs the survival of mice and delays the appearance of mouse mammary tumors. Clin Exp Metastasis.

[11] Loukinov D, Ghochikyan A, Mkrtichyan M, Ichim TE, Lobanenkov VV, Cribbs DH, Agadjanyan MG (2006). Antitumor efficacy of DNA vaccination to the epigenetically acting tumor promoting transcription factor BORIS and CD80 molecular adjuvant. J Cell Biochem.

[12] Mkrtichyan M, Ghochikyan A, Loukinov D, Davtyan H, Ichim TE, Cribbs DH, Lobanenkov VV, Agadjanyan MG (2008). DNA, but not protein vaccine based on mutated BORIS antigen significantly inhibits tumor growth and prolongs the survival of mice. Gene Ther.

[13] Pulaski BA, Ostrand-Rosenberg S, Coligan JE, Kruisbeek AM, Margulies DH, Shevach EM, Strober W (2001). Mouse 4T1 Breast Tumor Model. Current Protocols in Immunology.

[14] Monzavi-Karbassi B, Whitehead TL, Jousheghany F, Artaud C, Hennings L, Shaaf S, Slaughter A, Korourian S, Kelly T, Blaszczyk-Thurin M, Kieber-Emmons T (2005). Deficiency in surface expression of E-selectin ligand promotes lung colonization in a mouse model of breast cancer. Int J Cancer.

[15] Mkrtichyan M, Ghochikyan A, Davtyan H, Movsesyan N, Loukinov D, Lobanenkov V, Cribbs DH, Laust AK, Nelson EL, Agadjanyan MG (2011). Cancer-testis antigen, BORIS based vaccine delivered by dendritic cells is extremely effective against a very aggressive and highly metastatic mouse mammary carcinoma. Cell Immunol.

[16] Tukhvatulin AI, Gitlin II, Shcheblyakov DV, Artemicheva NM, Burdelya LG, Shmarov MM, Naroditsky BS, Gudkov AV, Gintsburg AL, Logunov DY (2013). Combined stimulation of Toll-like receptor 5 and NOD1 strongly potentiates activity of NF-kappaB, resulting in enhanced innate immune reactions and resistance to Salmonella enterica serovar Typhimurium infection. Infect Immun.

[17] Pulaski BA, Terman DS, Khan S, Muller E, Ostrand-Rosenberg S (2000). Cooperativity of Staphylococcal aureus enterotoxin B superantigen, major histocompatibility complex class II, and CD80 for immunotherapy of advanced spontaneous metastases in a clinically relevant postoperative mouse breast cancer model. Cancer Res.

[18] Bunt SK, Yang L, Sinha P, Clements VK, Leips J, Ostrand-Rosenberg S (2007). Reduced inflammation in the tumor microenvironment delays the accumulation of myeloid-derived suppressor cells and limits tumor progression. Cancer Res.

[19] Younos I, Donkor M, Hoke T, Dafferner A, Samson H, Westphal S, Talmadge J (2011). Tumor- and organ-dependent infiltration by myeloid-derived suppressor cells. Int Immunopharmacol.

[20] Ataullakhanov RI, Pichugin AV, Shishkova NM, Masternak TB, Malkina EY, Ulyanova LI (2005). Cellular mechanisms of immunomodulating action of the drug “Immunomax”. Immunologia.

[21] Ataullakhanov R, Tishchenko AL, Bauer HW, Grigoryan V, Shpot E, vor dem Esche U, Zgaga-Griesz A, Bessler WG (2010). Immunomax^R^ therapy to obtain relief in papilloma virus infections, prostatitis, and prostate carcinoma. J Mens Health.

[22] Lauzon NM, Mian F, Ashkar AA (2007). Toll-like receptors, natural killer cells and innate immunity. Adv Exp Med Biol.

[23] Moretta A (2002). Natural killer cells and dendritic cells: rendezvous in abused tissues. Nat Rev Immunol.

[24] Ferlazzo G, Tsang ML, Moretta L, Melioli G, Steinman RM, Munz C (2002). Human dendritic cells activate resting natural killer (NK) cells and are recognized via the NKp30 receptor by activated NK cells. J Exp Med.

[25] Gerosa F, Baldani-Guerra B, Nisii C, Marchesini V, Carra G, Trinchieri G (2002). Reciprocal activating interaction between natural killer cells and dendritic cells. J Exp Med.

[26] Schreibelt G, Tel J, Sliepen KH, Benitez-Ribas D, Figdor CG, Adema GJ, de Vries IJ (2010). Toll-like receptor expression and function in human dendritic cell subsets: implications for dendritic cell-based anti-cancer immunotherapy. Cancer Immunol Immunother.

[27] Kawamoto T, Ii M, Kitazaki T, Iizawa Y, Kimura H (2008). TAK-242 selectively suppresses Toll-like receptor 4-signaling mediated by the intracellular domain. Eur J Pharmacol.

[28] Matsunaga N, Tsuchimori N, Matsumoto T, Ii M (2011). TAK-242 (resatorvid), a small-molecule inhibitor of Toll-like receptor (TLR) 4 signaling, binds selectively to TLR4 and interferes with interactions between TLR4 and its adaptor molecules. Mol Pharmacol.

[29] Mai CW, Kang YB, Pichika MR (2013). Should a Toll-like receptor 4 (TLR-4) agonist or antagonist be designed to treat cancer? TLR-4: its expression and effects in the ten most common cancers. Onco Targets Ther.

[30] Ahmed A, Redmond HP, Wang JH (2013). Links between Toll-like receptor 4 and breast cancer. Oncoimmunology.

[31] Ahmed A, Wang JH, Redmond HP (2013). Silencing of TLR4 increases tumor progression and lung metastasis in a murine model of breast cancer. Ann Surg Oncol.

[32] Engelhardt R, Mackensen A, Galanos C (1991). Phase I trial of intravenously administered endotoxin (Salmonella abortus equi) in cancer patients. Cancer Res.

[33] Mackensen A, Galanos C, Engelhardt R (1991). Treatment of cancer patients with endotoxin induces release of endogenous cytokines. Pathobiology.

[34] A service of the U.S. National Institutes of Health, website: Clinicaltrials.gov **Identifier: NCT01585350, NCT01699347, NCT00010452, NCT00795977, NCT00291473, NCT01149902.**

[35] Cheever MA (2008). Twelve immunotherapy drugs that could cure cancers. Immunol Rev.

[36] Hurwitz AA, Yu TF, Leach DR, Allison JP (1998). CTLA-4 blockade synergizes with tumor-derived granulocyte-macrophage colony-stimulating factor for treatment of an experimental mammary carcinoma. Proc Natl Acad Sci U S A.

[37] Topalian SL, Hodi FS, Brahmer JR, Gettinger SN, Smith DC, McDermott DF, Powderly JD, Carvajal RD, Sosman JA, Atkins MB, Leming PD, Spigel DR, Antonia SJ, Horn L, Drake CG, Pardoll DM, Chen L, Sharfman WH, Anders RA, Taube JM, McMiller TL, Xu H, Korman AJ, Jure-Kunkel M, Agrawal S, McDonald D, Kollia GD, Gupta A, Wigginton JM, Sznol M (2012). Safety, activity, and immune correlates of anti-PD-1 antibody in cancer. N Engl J Med.

[38] Phan GQ, Yang JC, Sherry RM, Hwu P, Topalian SL, Schwartzentruber DJ, Restifo NP, Haworth LR, Seipp CA, Freezer LJ, Morton KE, Mavroukakis SA, Duray PH, Steinberg SM, Allison JP, Davis TA, Rosenberg SA (2003). Cancer regression and autoimmunity induced by cytotoxic T lymphocyte-associated antigen 4 blockade in patients with metastatic melanoma. Proc Natl Acad Sci U S A.

