# Supplementary material for: Targeting TLR-4 with a novel pharmaceutical grade plant derived agonist, Immunomax®, as a therapeutic strategy for metastatic breast cancer
Source: J Transl Med. 2014 Nov 29;12:322. doi: 10.1186/s12967-014-0322-y (PMC4261251; doi:10.1186/s12967-014-0322-y)
Supplement: Additional file 1: Figure S1. — The level of activated effector [NK cells (A), CD4+(B) and CD8+T cells (C)] or suppressor [MDSC (D) and Treg (E)] cells in the lungs of Immunomax® injected mice on day 20 post-surgery. Representative FACS images are presented. Figure S2. (A) Complete inhibition of 4T1 cell growth by splenocyte/4T1 co-culture in the presence of Immunomax®. (B) Activation of splenocytes isolated from 4T1 tumor bearing mice with Immunomax® inhibited the growth of splenic metastatic tumor cells. Figure S3. Flow cytometry gating strategy for CD4-, CD8-, CD19-, DX5+ NK cells in splenocytes. Figure S4. The gating strategy (A) and purity (B) of sorted population of mouse S-DCs. Purity of the sorted population was 97-98%. Figure S5. Purity of sorted splenic NK cell (A) and BM-DC (B) populations. Figure S6. A flow cytometry gating strategy to identify NK cells (CD45+, CD56+, CD16+) and representative histograms of CD69- expression by NK cells pre-incubated with or without Immunomax®. Figure S7. Human blood DC FACS-sorting (A) and gating (B) strategy. P-DC were identified as the lin1neg CD11cneg CD123high HLA-DRhigh, while M-DC as the lin1neg CD11chigh CD123neg HLA-DRhigh. (C) Purity of the sorted M-DC and P-DC. (D) Sensitivity of RT-PCR using the sorted DC. RT-PCR data for mRNA of hypoxanthine-guanine phosphoribosyl-transferase using different numbers of sorted DC per sample are represented. Figure S8. NF-κB activity measured as NF-κB-dependent SEAP reporter gene expression in HEK-Blue TLR null, 2, 3, 4, 5, 7, 8, 9 cells treated with Immunomax®. Intact cells were used as the negative control. TNF-α (10 ng/ml), Pam2CSK4 (1 μg/ml), poly I:C (10 μg/ml), LPS (1 μg/ml), flagellin (1 μg/ml), Imiquimod (1 μg/ml), CL097 (1 μg/ml), ODN 2007 (10 μg/ml) were used as positive controls. Results are expressed as the fold-increase in NF-κB-dependent SEAP reporter gene activity relative to intact untreated cells. [file 12967_2014_322_MOESM1_ESM.doc]

Additional file

**Targeting TLR-4 with a novel pharmaceutical grade plant derived agonist, *Immunomax®*, as a therapeutic strategy for metastatic breast cancer**

Anahit Ghochikyan, Alexey Pichugin, Alexander Bagaev, Arpine Davtyan, Armine Hovakimyan , Amir Tukhvatulin, Hayk Davtyan, Dmitry Shcheblyakov, Denis Logunov, Marina Chulkina, Anastasia Savilova, Dmitry Trofimov, Edward E. Nelson, Michael G. Agadjanyan, Ravshan I. Ataullakhanov

**
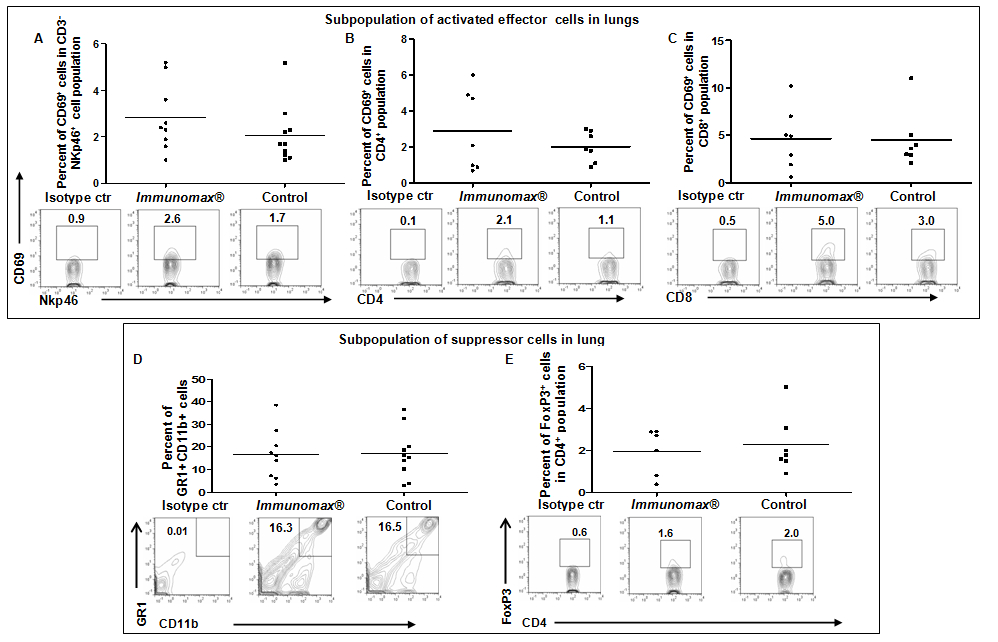
**

Figure S1. The level of activated effector [NK cells (A), CD4^+^(B) and CD8^+^T cells (C)] or suppressor [MDSC (D) and Treg (E)] cells in the lungs of Immunomax® injected mice on day 20 post-surgery. Representative FACS images are presented.

.

**A**

**B**


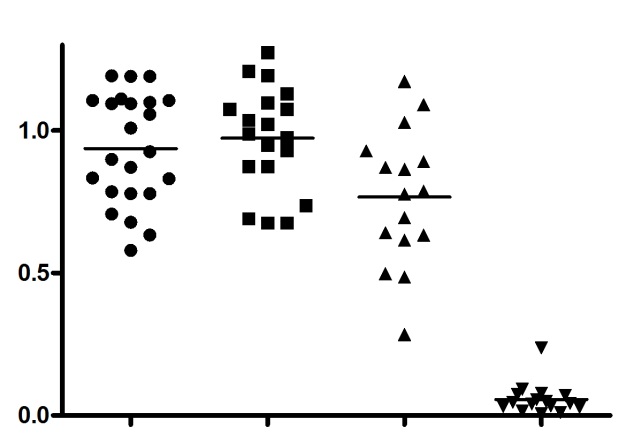


******

******

******

**Medium *Immunomax®***


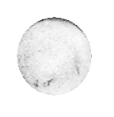

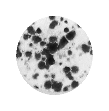

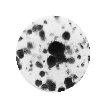

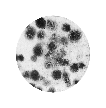


**4T1 (50 cells/well)**

**Splenocytes 5x10^4^/well**

***Immunomax@* 10µg/ml**

**+ + + +**

**- - + +**

**- + - +**

**Value of the color density of 4T1 colonies per well (normalized)**

**Value of the color density of 4T1 colonies per well (normalized)**


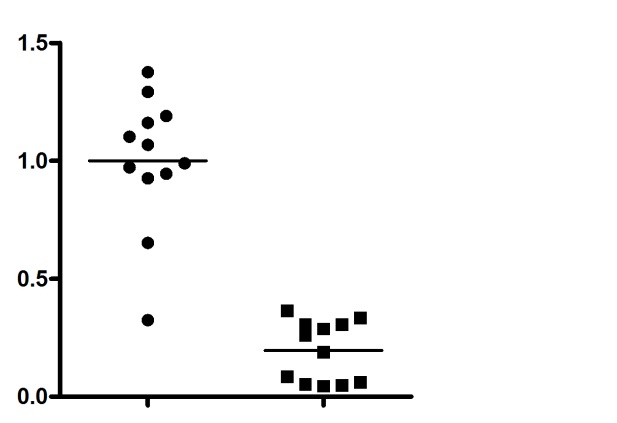


******

Figure S2. (A) Complete inhibition of 4T1 cell growth by splenocyte/4T1 co-culture in the presence of Immunomax®. (B) Activation of splenocytes isolated from 4T1 tumor bearing mice with Immunomax® inhibited the growth of splenic metastatic tumor cells.


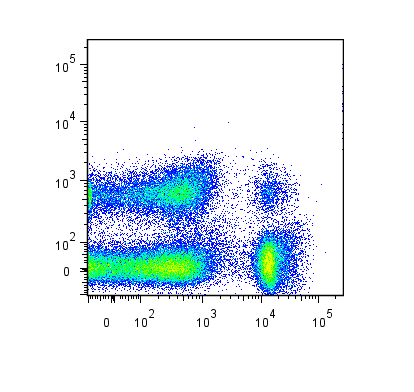

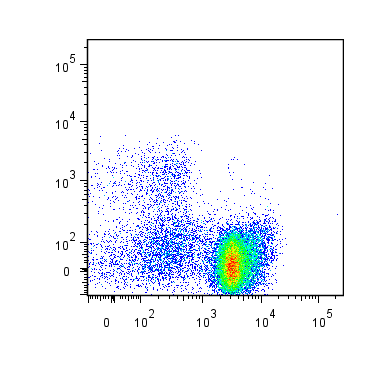


**CD4**

**CD8**

**50%**

**CD19**

**DX5**

**CD4- CD8- CD19- DX5+**

**3% of splenocytes**

**CD4- CD8-**

**Figure S3**. Flow cytometry gating strategy for CD4^-^, CD8^-^, CD19^-^, DX5^+^ NK cells in splenocytes.

**A**

**
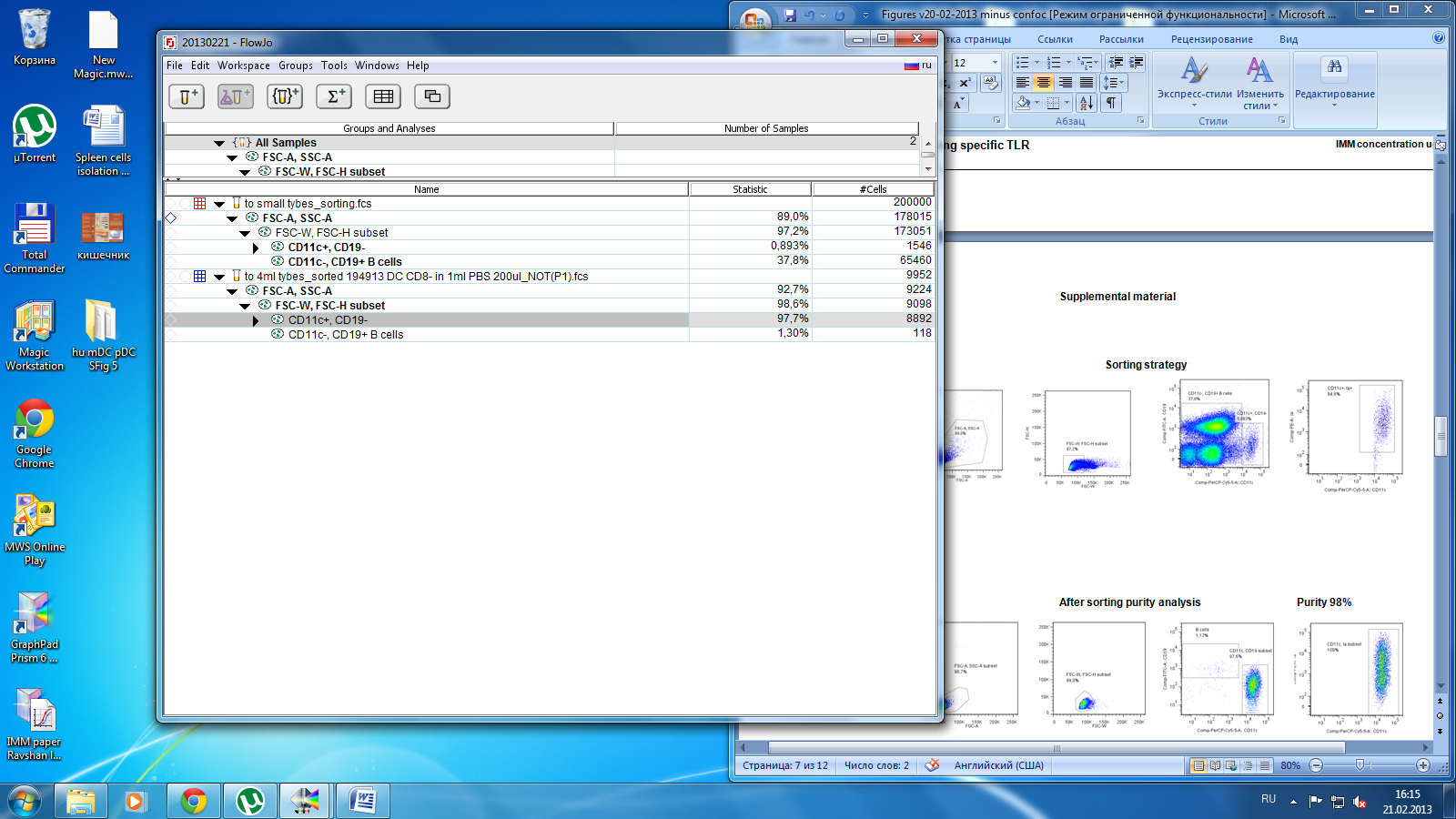
**

**Purity 97.7%**

**B**

**
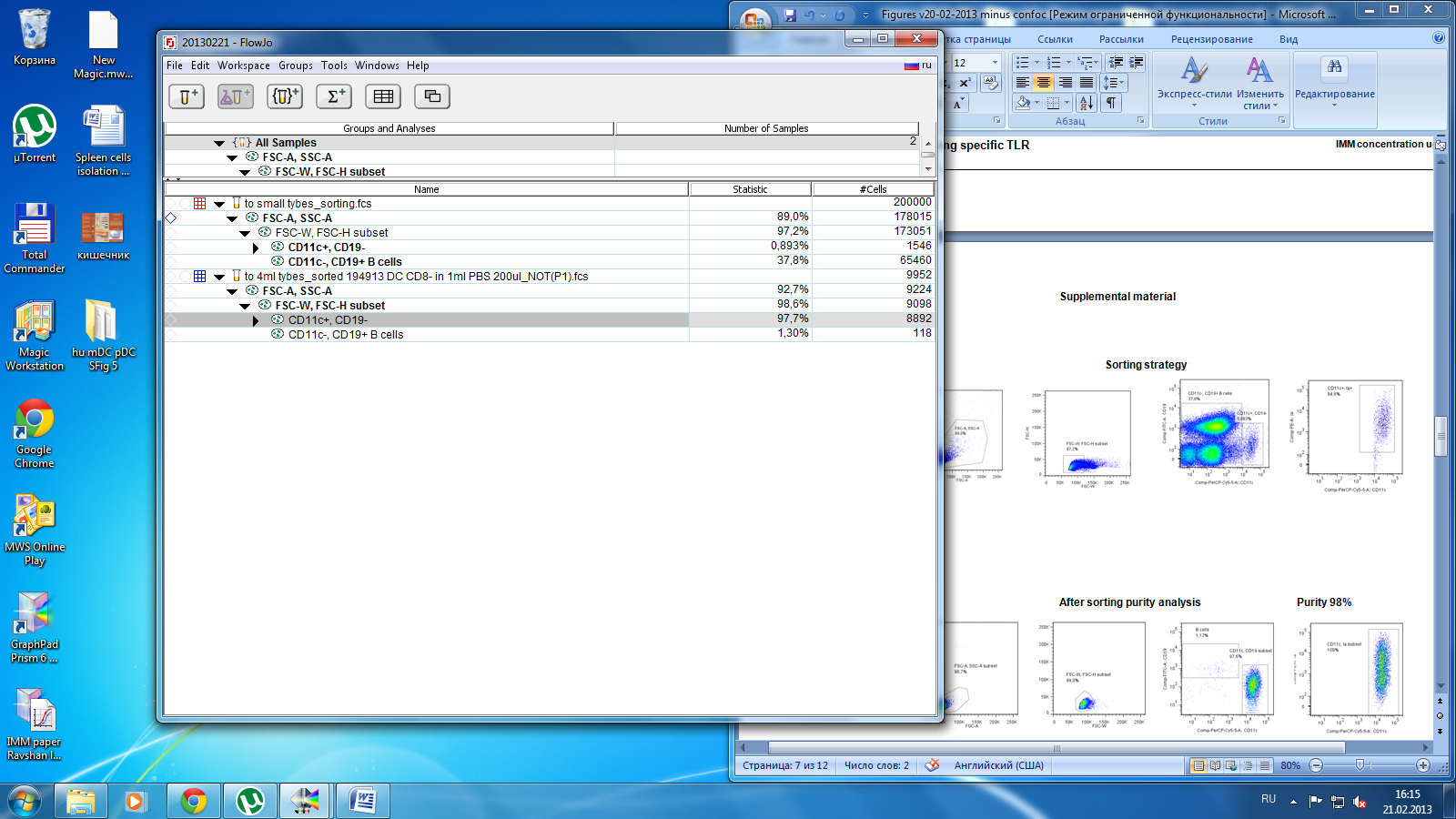
**

Figure S4. The gating strategy (A) and purity (B) of sorted population of mouse S-DCs. Purity of the sorted population was 97-98%.

**
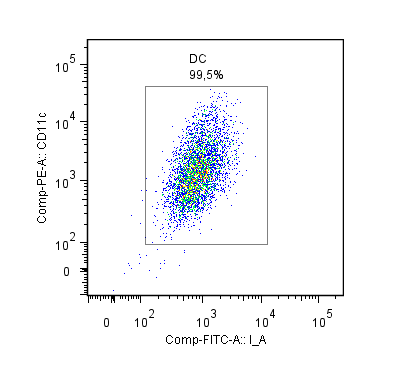
**

**CD11c**


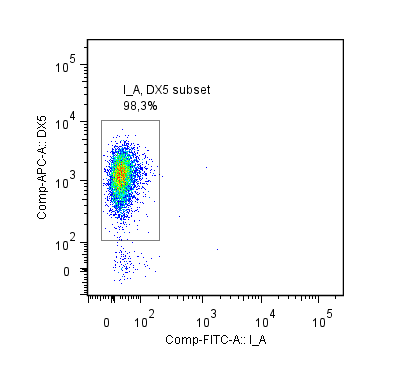


**I-A/E**

**DX5**

**98**%

**A**

**B**

**I-A/E**

**99%**

**Figure S5.** Purity of sorted splenic NK cell (A) and BM-DC (B) populations.

**
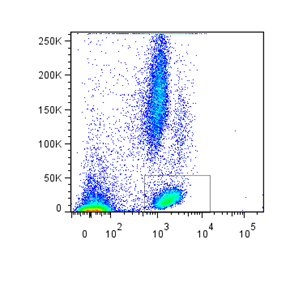
**


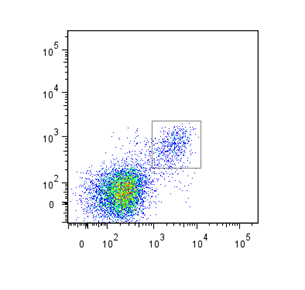


**CD45+ CD56 + CD16+**

**CD45+**

**CD16**

**CD56**


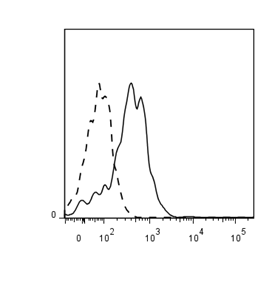


**CD45**

**SSC**

**Control**

**IMM**

**CD69**

Figure S6. A flow cytometry gating strategy to identify NK cells (CD45^+^, CD56^+^, CD16^+^) and representative histograms of CD69- expression by NK cells pre-incubated with or without Immunomax®.


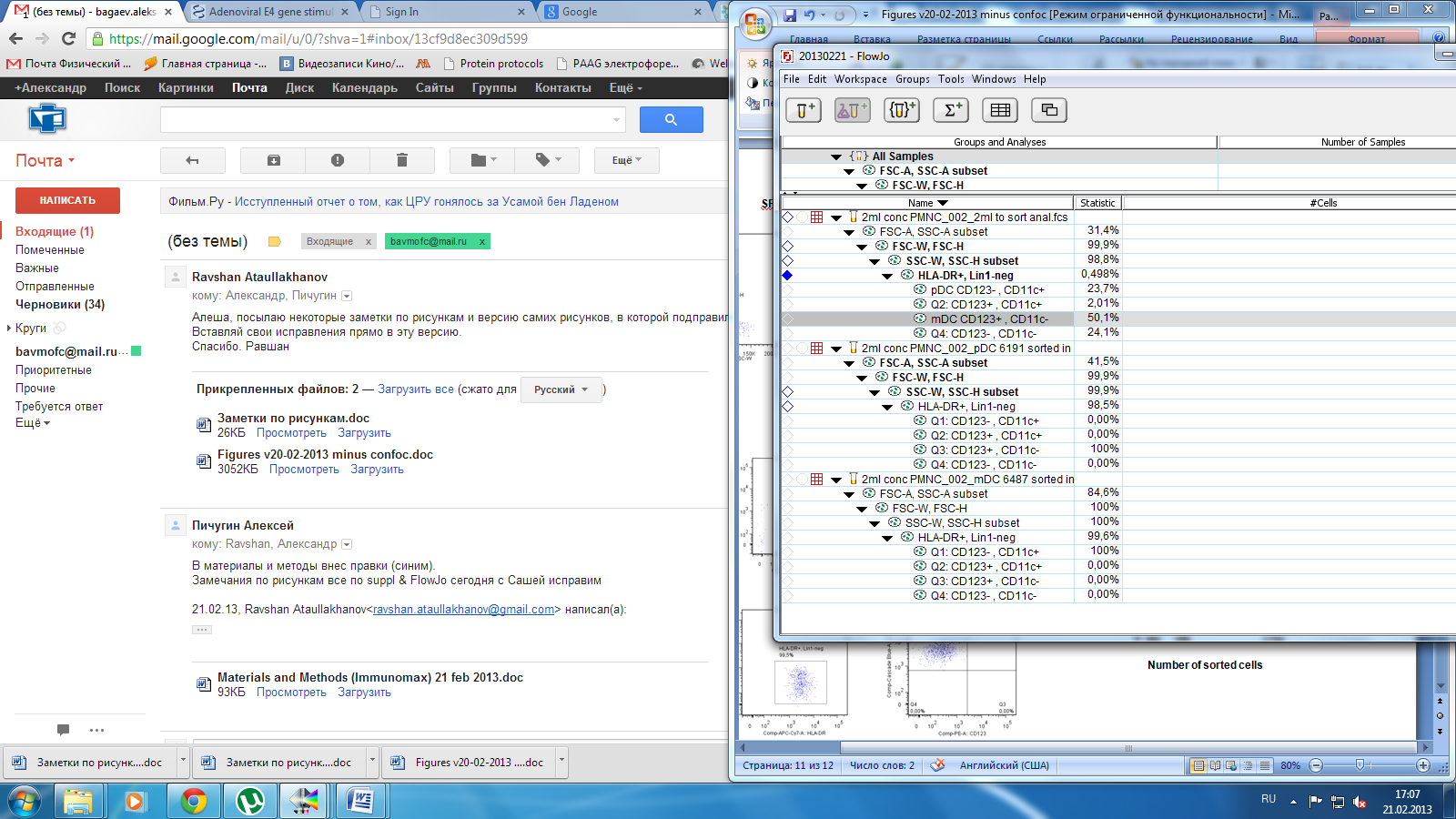


**Number of sorted cells**

**RT-PCR cycle**

**threshold (C_t_ )**

**A**

**M-DC Purity 99%**

**P-DC purity 98.5%**

**B**

**C**

**D**

Figure S7. Human blood DC FACS-sorting (A) and gating (B) strategy. P-DC were identified as the lin1^neg^ CD11c^neg^ CD123^high^ HLA-DR^high^, while M-DC as the lin1^neg^ CD11c^high^ CD123^neg^ HLA-DR^high^. (C) Purity of the sorted M-DC and P-DC. (D) Sensitivity of RT-PCR using the sorted DC. RT-PCR data for mRNA of hypoxanthine-guanine phosphoribosyl-transferase using different numbers of sorted DC per sample are represented.

**
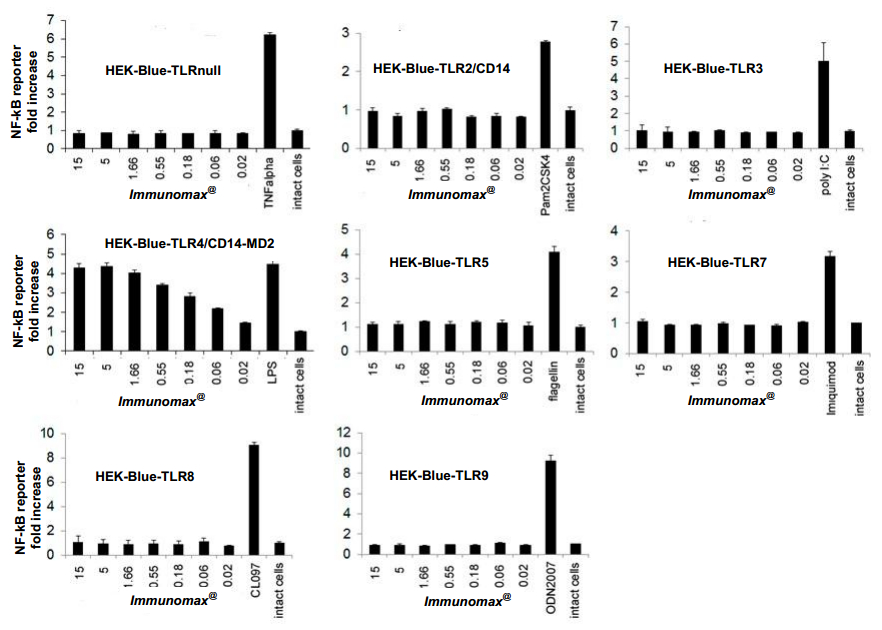
**

Figure S8. NF-κB activity measured as NF-κB-dependent SEAP reporter gene expression in HEK-Blue TLR null, 2, 3, 4, 5, 7, 8, 9 cells treated with Immunomax®. Intact cells were used as the negative control. TNF-α (10ng/ml), Pam2CSK4 (1 μg/ml), poly I:C (10 μg/ml), LPS (1 μg/ml), flagellin (1 μg/ml), Imiquimod (1 μg/ml), CL097 (1 μg/ml), ODN 2007 (10 μg/ml) were used as positive controls. Results are expressed as the fold-increase in NF-κB-dependent SEAP reporter gene activity relative to intact untreated cells.
